# Pilot study on RECAF (Receptor for Alpha-Fetoprotein) expression in pediatric acute leukemia: insights from flow cytometry analysis

**DOI:** 10.1186/s43046-026-00404-4

**Published:** 2026-09-02

**Authors:** Soha Raouf Youssef, Mohamed Hany Mohammady, Shams ElDoha Galal ElDin Zaiema

**Affiliations:** Ain shams University, Cairo, Egypt

**Keywords:** RECAF, Pediatric acute myeloid leukemia, Pediatric acute B-lymphocytic leukemia, Acute T-lymphocytic leukemia, Flow cytometric immunophenotyping (FCI), Emerging leukemic biomarkers

## Abstract

**Background:**

Acute leukemia is the most common cancer in children, highlighting the importance of early diagnosis. RECAF is an AFP receptor that is abnormally re-expressed in various malignant tumors, serving as a promising biomarker for tumors.

**Aim:**

This study aimed to assess the clinical utility of RECAF expression by flow cytometry immunophenotyping in pediatric de novo acute leukemia patients.

**Methods:**

Our study was conducted in 80 pediatric patients with acute leukemia, comprising 40 newly diagnosed ALL patients, 40 newly diagnosed AML patients, and 40 age- and sex-matched non-malignant control subjects. Flow cytometric analysis for the Acute Leukemia panel and RECAF expression was performed on all subjects, along with other laboratory and clinical parameters.

**Results:**

Our study revealed that RECAF expression was significantly higher in patients with acute leukemia than in controls. We also established a cutoff of ≥ 13.31% for positive RECAF expression by flow cytometry to distinguish RECAF-positive acute leukemia patients from FECAF-negative patients and normal controls.

**Conclusion:**

Our findings suggest that RECAF could be an important biomarker for differentiating leukemic blasts and may become a useful tool for monitoring measurable residual disease in the future. Such advancements have the potential to improve clinical outcomes and enhance patient monitoring and management for this high-risk group.

**Supplementary Information:**

The online version contains supplementary material available at https://doi.org/10.1186/s43046-026-00404-4.

## Introduction

Pediatric leukemia has the highest incidence rate of 3–4 cases per 100,000 in patients under 18 years old, and its incidence is increasing. More than 90% of these cases are classified as acute leukemia [1]. Acute lymphoblastic leukemia (ALL) is the most common type of cancer found in children. This condition arises from chromosomal abnormalities and genetic changes that affect the differentiation and growth of lymphoid progenitor cells. Most cases are classified as B-cell precursor, while approximately 15% express markers of T-cell progenitors [2]. Acute myeloid leukemia (AML), which is less commonly reported in the pediatric population, is characterized by the infiltration of the bone marrow, blood, and other tissues by clonal, abnormally differentiated, poorly differentiated, or immature hematopoietic cells of various myeloid lineages. This results in ineffective erythropoiesis and bone marrow failure [3]. In Egypt, ALL occurs more frequently than AML in pediatrics, accounting for about 20% of pediatric malignancies and approximately 4 cases per 100,000 children [4]. 

The diagnosis of AL is generally made based on clinical presentation, morphologic identification of leukemic blasts in preparation of peripheral blood and bone marrow stained with Wright—Giemsa, immunophenotypic features, and cytogenetic and molecular genetic mutations [5]. Flow cytometric immunophenotyping is a cornerstone of the diagnostic workup of pediatric AL. It enables immunological identification and sub-classification of blasts by assessing cellular antigen expression and provides a footprint for monitoring residual disease post-therapy [6]. It also enables the detection and follow-up of minimal residual disease (MRD) post-therapy using the leukemia-associated immunophenotype (LAIP), a subtype-specific, patient-tailored approach [7, 8]. 

Alpha-fetoprotein is a pivotal protein synthesized during embryonic development [9]. Its high levels in fetal serum suggest that it can stimulate the proliferation and differentiation of various cell types, including lymphoid and epidermal cells, fibroblasts, hepatocytes, and ovarian and uterine cells, as has long been demonstrated [10]. 

RECAF, the receptor for circulating alpha-fetoprotein (AFP), plays a crucial role during fetal development by facilitating the uptake of AFP into various tissues, an essential step in their growth and differentiation. Notably, RECAF expression declines markedly after birth, underscoring its specific importance in prenatal biology [9]. Surprisingly, many recent in vitro and in vivo studies have shown that some malignant tumors regain the ability to take up AFP by expressing RECAF, including ovarian, hepatic, and testicular carcinomas [11–15]. Also, recent research by Sedky et al. (2020) reported distinct RECAF expression patterns in tissue biopsies from patients with B non-Hodgkin lymphoma/chronic lymphocytic leukemia (B-NHL/CLL) and other hematological malignancies [16]. 

Although non-specific, these recent findings highlight the potential of RECAF, when combined with other tissue-specific markers, to increase sensitivity for early relapse detection and follow-up of malignant tumors, in which the prevalent tumor biomarker may be elevated in benign lesions [17]. It could also serve as a future target therapy in these malignancies [18]. 

Building on these prior studies, we designed our study to assess the clinical significance of RECAF expression, as measured by flow cytometric immunophenotyping (FCI), in pediatric patients diagnosed with acute leukemia. This investigation also explores potential correlations between RECAF expression and other markers in leukemia panels, as well as with key clinical and laboratory parameters.

## Patients and methods

### Patients

**Study Design and Setting: This pilot clinical study was conducted on 120 candidates for bone marrow aspiration from 2023 to 2025, enrolled in the Pediatric Hematology/Oncology Department at Ain Shams University Hospitals. Data sources: Medical history data were collected from hospital files. Inclusion criteria: All subjects were under 18 years of age. ** Eighty (*n* = 80) newly diagnosed acute leukemia (AL) patients (40 ALL patients and 40 AML patients) were diagnosed based on WHO HAEM5 and ICC morphological, IPT, and cytogenetic criteria. Age- and sex-matched controls were 40 subjects admitted for BM examination for causes other than hematological or non-hematological malignancies (e.g., patients diagnosed with immune thrombocytopenia (ITP) or hypersplenism. The exclusion criteria for candidate subjects included: patients with solid tumors; patients with other hematological malignancies; and Exposure to leukemogenic therapies or agents.

### Methods

**Sampling Method: Bone marrow (BM) aspirate samples were obtained aseptically from each patient and used for Leishman and myeloperoxidase (MPO) staining smears, assessment of FCI panels for acute leukemia, RECAF expression analysis using a Beckman Coulter NAVIOS flow cytometer, and cytogenetic analysis. Peripheral blood samples were collected for complete blood count (CBC) and biochemical profile analysis.

**Fig. 1:**
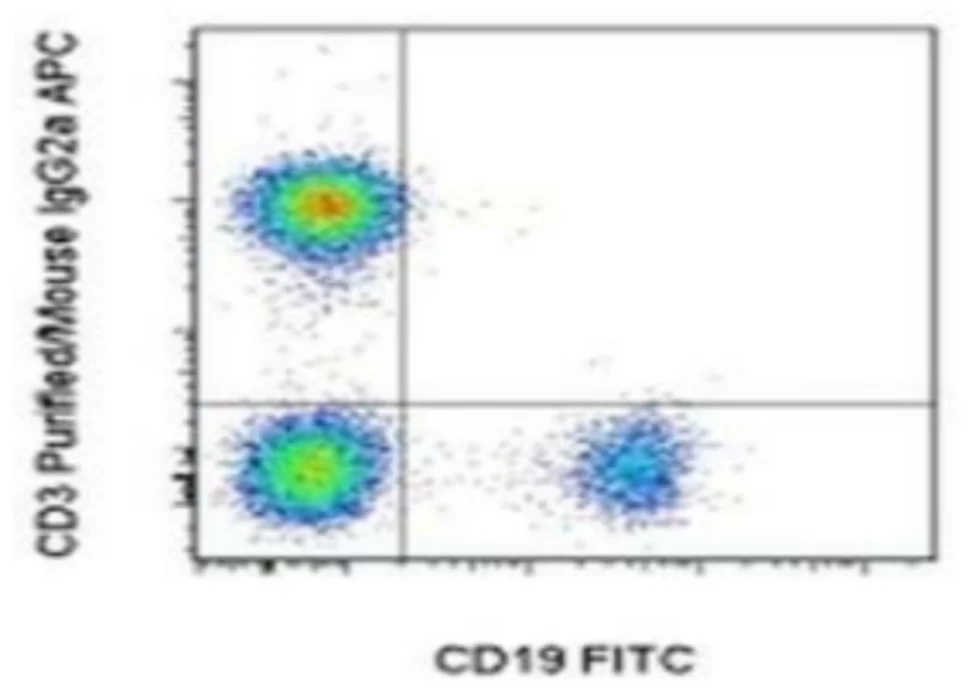
Mouse IgG2a Secondary Antibody on cells within the lymphocyte gate was analyzed by flow cytometry. Normal human peripheral blood cells were stained with Anti-Human CD19 FITC and Anti-Human CD3 Purified, followed by staining with Anti-Mouse IgG2a APC (negative staining in normal lymphocytes)

**Fig. 2:**
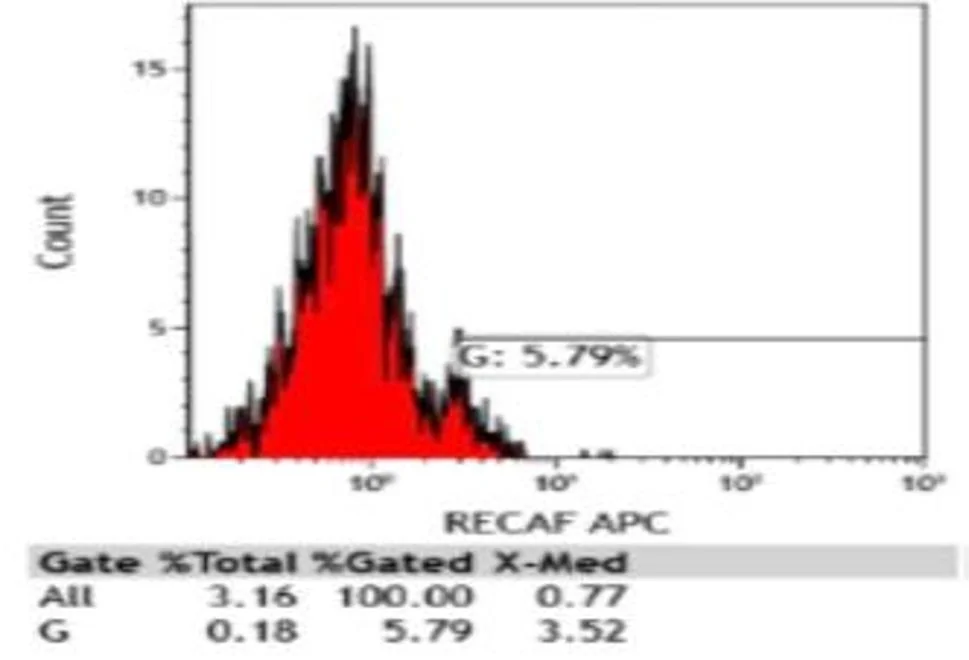
Showing Negative staining of Mouse IgG2a Secondary Antibody by flow cytometry (without Primary Ab) on Blast cells in AL patients

**Clinical and Laboratory Procedures for Participants:

1. Clinical Assessment, Radiological Investigation, and Routine Diagnostic Workups: The evaluation primarily focused on leukemia-associated symptoms, visceralomegaly (especially hepatosplenomegaly (HSM)), central nervous system (CNS) or testicular involvement, and lymphadenopathy. CBC and examination of Leishman-stained peripheral blood (PB) and Bone marrow (BM) smears were performed to assess blast count. Biochemical profile tests, including lactate dehydrogenase (LDH) and uric acid, were also performed.
2. Flow Cytometric Analysis:
  a Flow cytometric analysis for the leukemia panel:
    i Assessment was conducted on BM using a Beckman Coulter Navios flow cytometer. Staining methods included direct staining for surface immunophenotypic markers (lysing without washing) and intracellular staining. Evaluated markers included: common progenitor markers (CD34 FITC, HLA-DR FITC), myeloid lineage markers (CD13 PE, CD33 PE, MPO FITC), B cell lymphoid markers (CD10 PE, CD19 PE, CD20 FITC, CD79a PE), and T cell lymphoid markers (CD2 PE, CD5 FITC, CD7 FITC).
    ii Flow cytometry data interpretation: Marker positivity was defined as ≥20% of leukemic cells expressing the marker, except for MPO, cCD3, and TDT, where positivity was defined as ≥10%.

b Flowcytometric analysis for RECAF expression: Surface RECAF expression was assessed by flow cytometry using primary and secondary monoclonal antibodies.
  i Primary Ab (AFP Receptor Antibody, 2B8) (mouse monoclonal anti-AFP receptor) (Santa Cruz Biotechnology, CA, USA):
    Description: This is a high-quality monoclonal AFP receptor antibody suitable for detecting the AFP receptor protein of human origin. It is of the IgG2a isotype. The vial contains 100 µg of IgG2a in 1.0 ml of PBS with < 0.1% sodium azide and 0.1% gelatin. Storage condition: Stored at 4°C without freezing.
    The primary antibody was titrated at 1:20, using 10 µL per sample, since sample cell counts were maintained between 5,000 and 10,000 cells/µL, with 50 µL of sample added per tube. Events were acquired until 50,000 events per tube.
  ii Secondary Ab (Rat Anti-Mouse IgG2a secondary antibodies, APC) (Invitrogen/Thermo Fisher Scientific):
    Description: The monoclonal antibody m2a-15F8 recognizes mouse IgG2a antibodies and was used as a secondary reagent for flow cytometry detection of RECAF expression. It does not recognize other mouse isotypes, nor does it cross-react with rat IgG2a or other rat isotypes. The antibody amount (µg) required to stain a cell sample to a final volume of 100 µL was determined empirically, with cell numbers ranging from 10^5 to 10^8 per test. The antibody was carefully titrated for optimal RECAF detection. Excitation: 633-647 nm; Emission: 660 nm; Laser: Red; Filtration: 0.2 µm post-filtered. Storage condition: Stored at 4°C without freezing. Product images for Rat anti-Mouse IgG2a Secondary Antibody, APC are shown in Fig. 1.
    Negative control: Mouse IgG2a secondary antibody staining (without primary antibody) on blast cells from AL patients, demonstrating secondary-only background, is shown in Fig. 2.
  iii The FMO controls were skipped because we used only three colors, keeping the RECAF assessment panel small, and chose fluorochromes with minimal spectral overlap (spillover). The APC-labeled Rat Anti-Mouse IgG2a secondary antibody gave a strong signal and was analyzed alongside the monoclonal antibodies for CD45 (dim signal, PC5-labeled) and CD34 (ECD-labeled). These helped identify cell populations and reduce spillover.
  iv Fc block was unnecessary because interference was expected mainly from myeloid cells, which were excluded by gating on blast cells using CD45/SSC. When blast cells were CD34 positive, further gating on CD34-positive blast cells was performed. An isotype-matched control was used in initial trials to ensure elimination of nonspecific binding.
  v Compensation, daily quality control, and data analysis were performed according to laboratory SOPs. Access to materials was restricted to authorized personnel. The lab is ISO 15189 accredited for relevant panels (CAB ref. 0513002).
  vi Pre-analytical variables included analyzing a fresh EDTA sample or tube, transferred to the lab within an hour of collection. The lysis protocol employed was an in-house ammonium chloride solution following a stain-lyse-wash protocol. No viability dye was used, and the sample was not stored post-analysis. Additionally, no relevant steroids or cytoreduction medication were administered before sampling.
  vii Flow cytometry data interpretation: For RECAF expression, we considered expression in ≥13.31 % of the sample’s cells in the "blast gate" (defined by dim CD45 and low side scatter) sufficient to confer positivity (as guided by ROC analysis in the results section).

4 Complete Hematological Remission (CHR) on day 28 was defined as having less than 3% circulating blasts, bone marrow blasts below 5%, no extramedullary disease, an absolute neutrophil count (ANC) of at least 1.0 × 10^9/L, and a platelet count of at least 100 × 10^9/L.
5 Karyotyping and FISH Analysis: to detect recurrent cytogenetic abnormalities. **Statistical methods: IBM SPSS version 23 was used to analyze the data. Normal distribution was assessed using the Shapiro-Wilk test. The Kruskal-Wallis test was used for non-parametric data. Quantitative data were presented as means ± standard deviations and ranges for parametric distributions, and as medians with interquartile ranges (IQRs) for nonparametric distributions. Qualitative data were shown as percentages and counts. Comparisons between groups utilized the Chi-Square Test or Fisher's exact test, with the independent t-test for parametric quantitative data and the Mann-Whitney test for non-parametric data. The receiver operating characteristic (ROC) curve identified optimal cutoff points, sensitivity, specificity, positive and negative predictive values, and the area under the curve (AUC). The significance level was set at P < 0.05.

## Results

A Describing and comparing demographic, clinical, and laboratory data between the control and patient groups (shown in Table 1 in the Supplementary data)

**Fig. 3:**
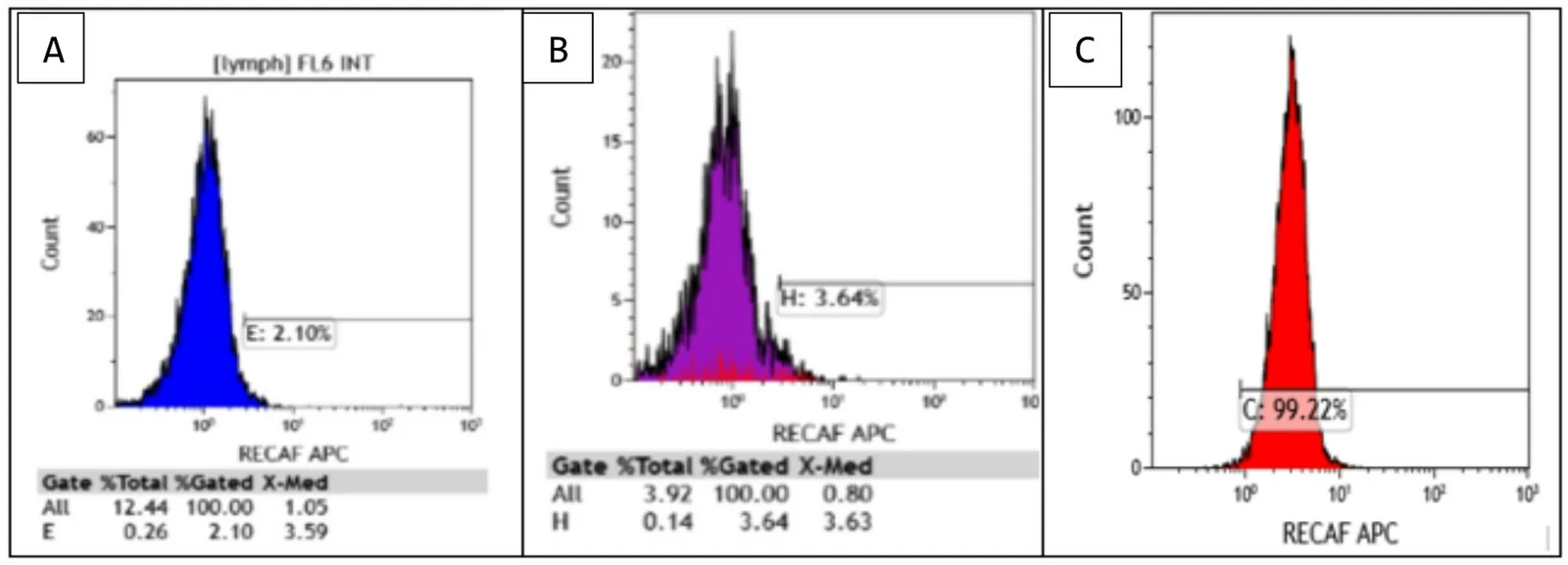
Comparison of RECAF% by flow cytometry between the control and patient groups; (**A**) shows negative RECAF expression on normal lymphocytes; (**B**) shows negative RECAF expression on normal Hematogones; and (**C**) shows positive RECAF expression in AL patients’ blast cells

**Fig. 4:**
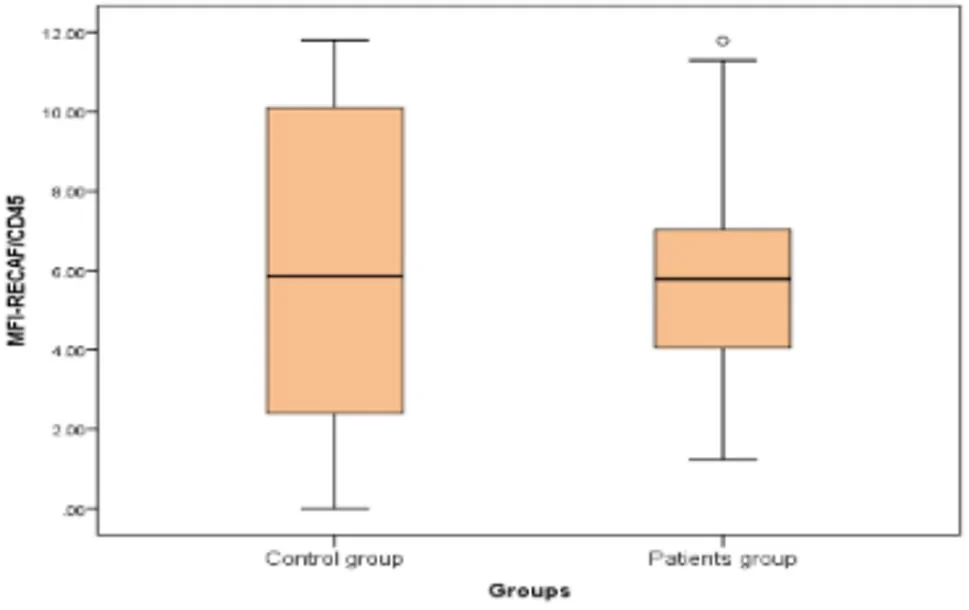
Comparison of RECAF-MFI by flow cytometry between the control and AL patient groups

- The control group’s age range was 2 to 13 years, with a median (IQR) of 12 (7–13) years, while the patient group’s age ranged from 1 to 17 years, with a median (IQR) of 12 (7–13) years.
- Clinical examination showed HSM in 36 out of 80 patients and 4 out of 40 controls.
- Comparing PB and BM findings between control and patient groups revealed a highly significant increase (*P* < 0.01) in Hb% in the control group (mean = 12.52 g/dl) compared to the patient group (mean = 8.04 g/dl). Additionally, the mean blast percentages in PB and BM in the control group (0% and 1.95%) were significantly lower (*P* < 0.01) than in the patient group (54.02% and 76.4%).
- The patient group showed a highly significant increase (*P* < 0.01) in the percentages of RECAF expression on blast cells as measured by flow cytometry, compared to controls. However, there was no significant difference in RECAF-MFI (expression intensity) on blast cells between the groups (*P* > 0.05) (Figs. 3 and 4).
- No other parameters showed significant differences.

**Fig. 5:**
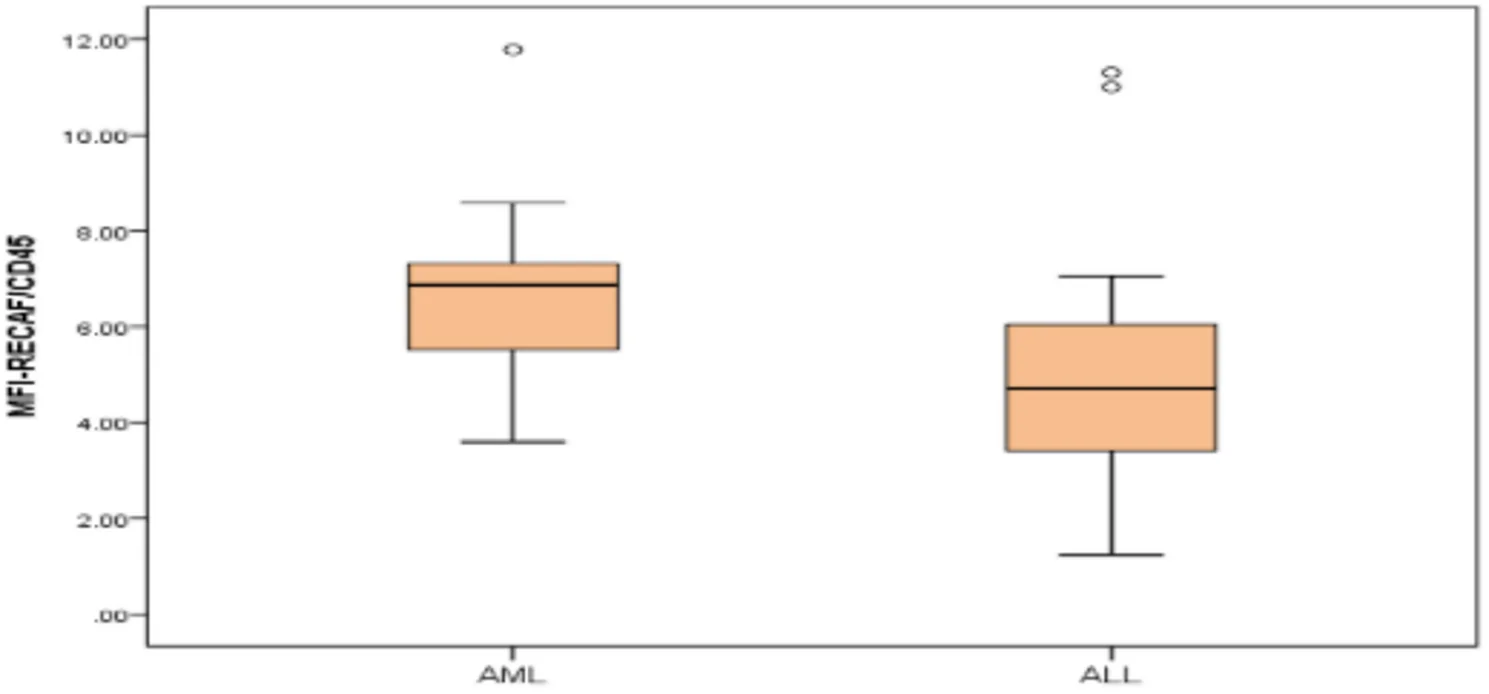
Comparison of RECAF-MFI by flow cytometry between the AML and ALL patient groups

B Describing and comparing demographic, clinical, and laboratory data in the AML vs. ALL sub-groups (shown in the Supplementary data Table 2):

1. There was no significant difference regarding demographic, clinical, and lab data, together with CD34 expression by flow cytometry and CHR on day 28, between the AML and ALL groups, except for the age range, which was higher in the AML group (P-value < 0.05), and the BM blast count was higher in the ALL group(P-value < 0.01).
2. There was no significant difference regarding the percentages of RECAF expression on blast cells as measured by flow cytometry (*P* > 0.05) in the AML vs. ALL sub-groups.
3. The MFI of RECAF expression showed a statistically significant increase (P-value < 0.05) in the AML group compared to the ALL group (Fig. 5).

C Correlation of the RECAF% expression and MFI in the CD45 Blast gate with other flowcytometric and laboratory data in all patients’ groups (Table 1)

**Table 1:** Correlation between the RECAF% percentage and MFI in the CD45/blast gate with other Flowcytometric and laboratory data in all patients’ groups:

All patient groups	RECAF%		RECAF MFI	
	*r*	*P*-value	*r*	*P*-value
RECAF%	–	–	-0.199	0.217
RECAF MFI	-0.199	0.217	–	–
Age (years)	-0.023	0.890	-0.021	0.896
CD34 (%)	0.258	0.107	0.085	0.600
HLA-DR (%)	0.143	0.378	0.127	0.436
TDT (%)	0.144	0.544	-0.115	0.630
CD117(%)	0.223	0.168	**0.393**	**0.012**
MPO (%)	0.231	0.151	**0.355**	**0.025**
CD13(%)	**0.357**	**0.024**	0.248	0.123
CD33(%)	0.260	0.105	0.223	0.166
CD64 (%)	**0.434**	**0.005**	**0.340**	**0.032**
CD11C (%)	**0.404**	**0.010**	0.111	0.495
CD19 (%)	-0.237	0.141	-0.147	0.367
CD79a (%)	-0.076	0.643	-0.250	0.119
CD10(%)	-0.232	0.149	-0.249	0.121
CD20(%)	0.036	0.827	-0.150	0.357
CD7(%)	-0.017	0.918	0.110	0.498
CD2(%)	0.027	0.868	-0.001	0.996
CD5(%)	0.201	0.215	0.048	0.770
CD3(%)	-0.017	0.918	0.110	0.498
TLC (10^9^/L)	0.032	0.846	0.015	0.929
Hb (g/dl)	-0.113	0.486	-0.308	0.053
Platelets (10^9^/L)	0.107	0.511	-0.083	0.611
PB blast%	-0.070	0.666	-0.205	0.205
BM blasts%	-0.131	0.421	-0.325*	**0.041**

*P*-value > 0.05: Non-significant; *P*-value < 0.05: Significant; *P*-value < 0.01: Highly significant, *Spearman correlation coefficient, Significant values are provided in bold

**Fig. 6:**
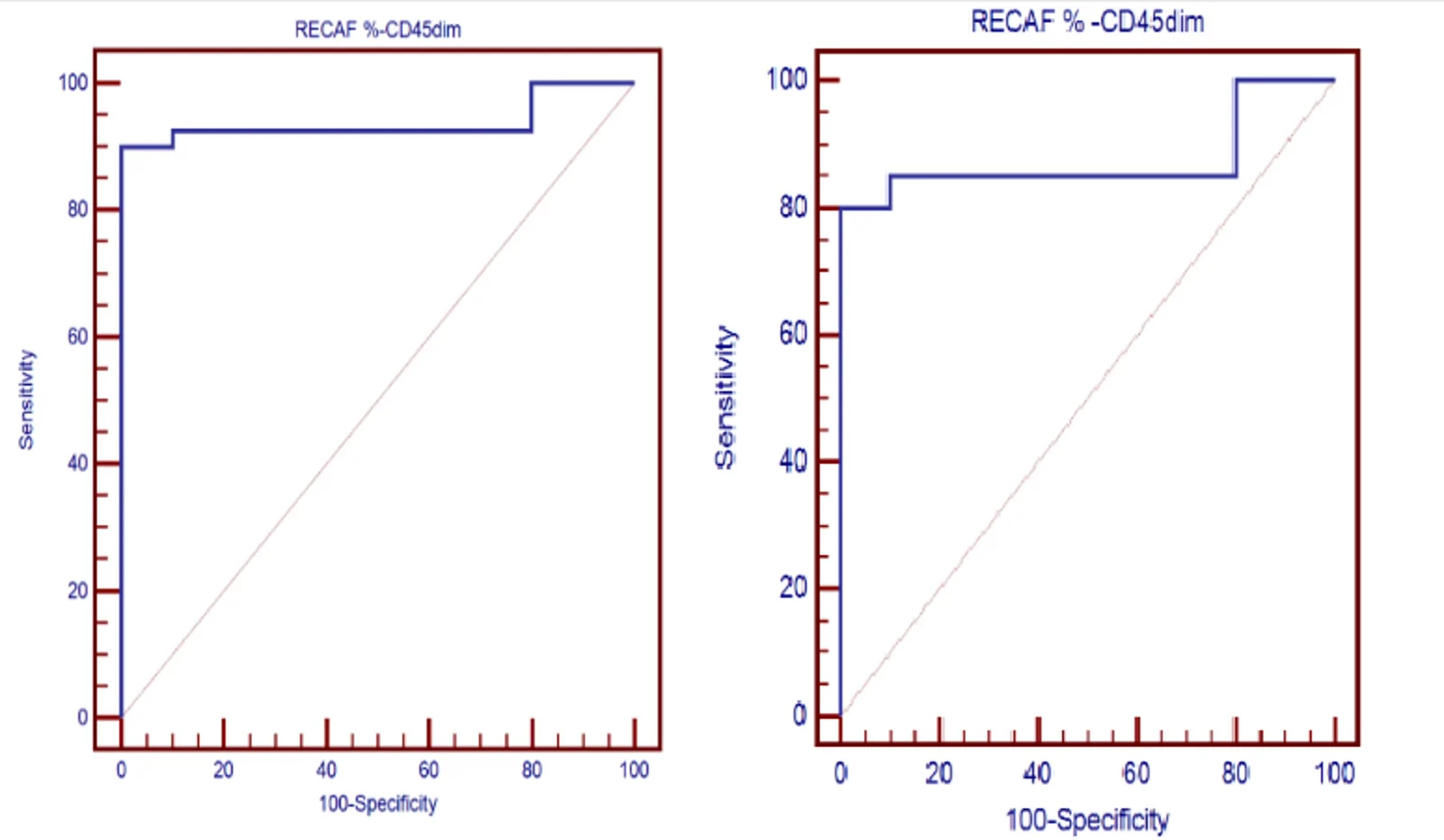
ROC curve analysis to set a positivity cutoff for RECAF expression; Control vs. All Patients (**a**), and ALL vs. Controls (**b**)

**Fig. 7:**
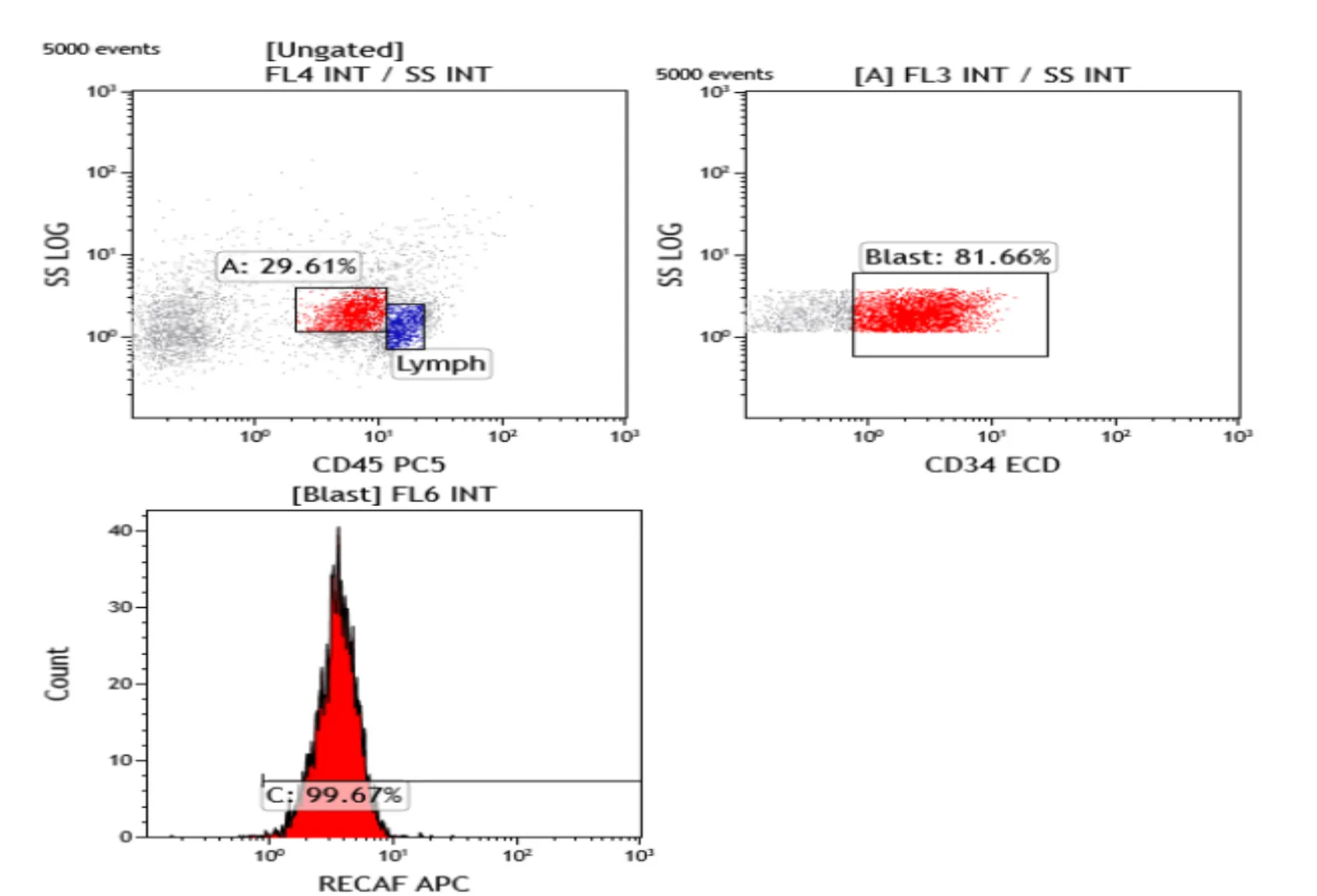
Positive RECAF expression in AML patients with CD34-positive blast cells

**Fig. 8:**
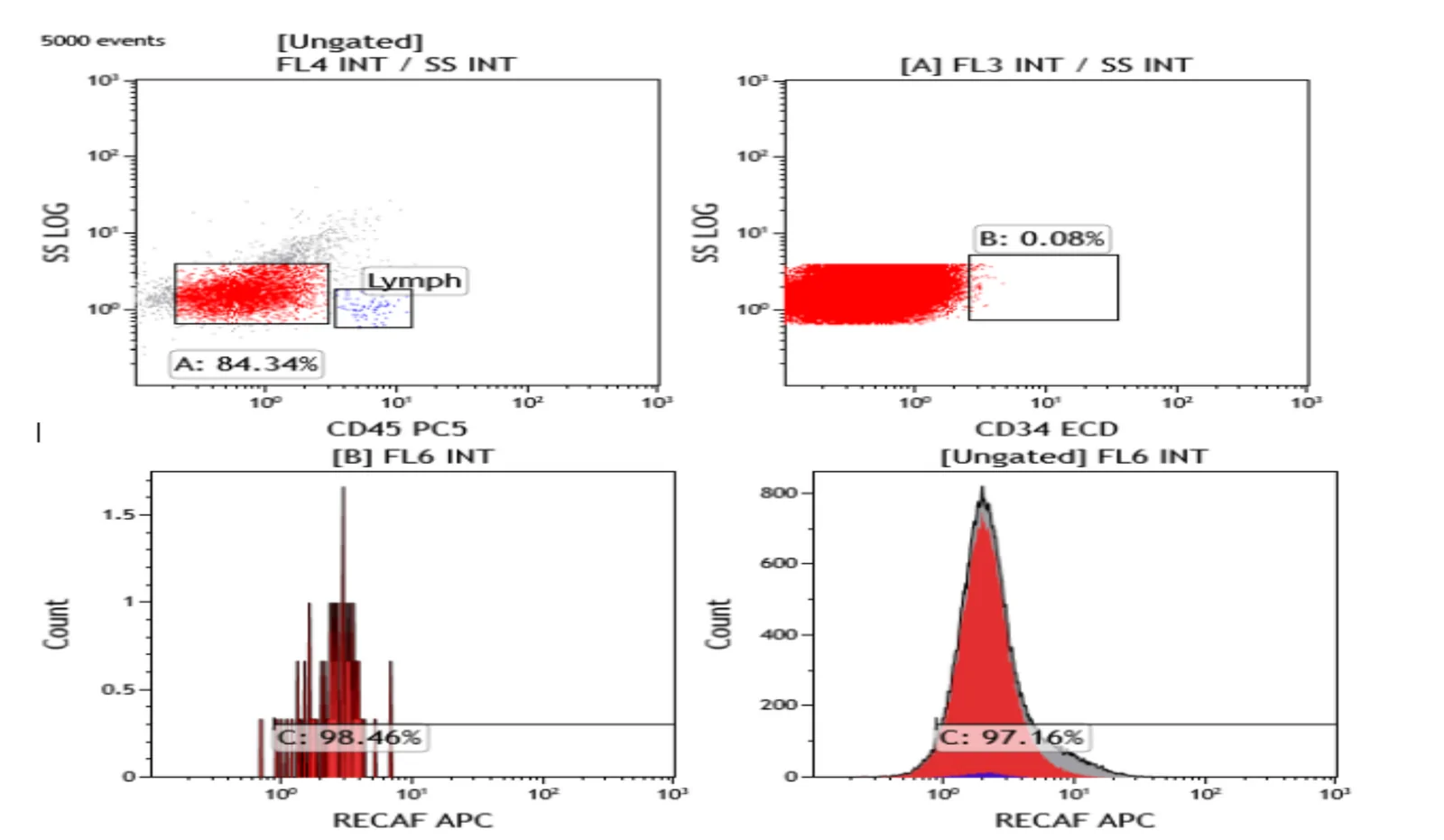
Positive RECAF expression in B-ALL patients with CD34-negative blast cells

D Multivariate linear regression analysis for factors associated with RECAF% and RECAF MFI among the studied patients (shown in the Supplementary data Table 3&4)
  Multivariate linear regression analysis identified CD13 as the only independent factor significantly associated with RECAF%, demonstrating a positive association (β = 0.314, B = 0.223, p = 0.040). In contrast, neither CD64 (β = 0.030, B = 0.026, p = 0.901) nor CD11c (β = 0.280, B = 0.365, p = 0.198) showed a statistically significant association with RECAF%.
  Multivariate linear regression analysis identified CD117 as the only independent factor significantly associated with RECAF-MFI, showing a positive association (β = 0.305, B = 0.017, p = 0.041). In contrast, MPO (β = 0.117, B = 0.006, p = 0.710), CD64 (β = 0.250, B = 0.018, p = 0.233), and bone marrow blasts percentage (β = −0.152, B = −0.016, p = 0.387) were not significantly associated with RECAF MFI.
E Studying the discriminative utility of RECAF expression between different groups (set a positivity cutoff) (Table 2 & Fig. 6):
F Comparing demographic, clinical, laboratory, and IPT data between RECAF negative (<13.31%) and positive (≥13.31%) acute leukemia patient subgroups (Table 3 & Figs. 7 and 8):
  Notably, all AML and T-ALL patients were positive for RECAF, whereas B-ALL patients were positive or negative.
  Positive RECAF expression in AL patients is shown in Fig. 5 (AML with CD34-positive blasts) and in Fig. 6 (ALL with CD34-negative blasts).
G Comparing demographic, clinical, laboratory, and IPT data between RECAF negative and positive B-ALL patients (Table 4):
H Demonstration of the relationship between CD34 and RECAF expression in our study (Table is shown in the Supplementary data):
  All CD34-positive acute leukemia patients tested positive for RECAF expression (100.0%); meanwhile, CD34-negative patients tested positive or negative for RECAF.
  RECAF-negative Acute leukemia patients tested negative for CD34. Meanwhile, RECAF-positive acute leukemia patients were tested for CD34, with some testing positive and others negative.

**Table 2:** The discriminative utility of RECAF expression between different groups

Groups	Control vs. All Patients	AML vs. Controls	ALL vs. Controls
Cutoff	≥ 13.31%	≥ 13.31%	≥ 13.31%
AUC	0.937	1.000	0.875
Sensitivity	90%	100%	80%
Specificity	100%	100%	100%
+PV	100%	100%	100%
-PV	71.4%	100%	71.4%

ROC curve analysis involving the maximization of the Youden index (J)

**Table 3:** Describing and comparing flowcytometric markers in all RECAF negative (< 13.31%) vs. positive (≥ 13.31%) acute leukemia patients’ sub-groups

All patient groups (No = 80)		RECAF%		*P*-value
		< 13.31% (Negative)	≥ 13.31% (Positive)	
		No. = 8	No. = 72	
Age (years)	Median (IQR)	11.5 (8–13.5)	11 (5–14.5)	0.982
	Range	6–14	1–17	
Sex	Female	2 (25.0%)	24 (33.3%)	0.736
	Male	6 (75.0%)	48 (66.7%)	
FAB subtype for AML (No. =40)	M0		2 (2.5.0%)	
	M1/M2		20 (25.0%)	
	M4/M5		10 (12.5%)	
	M3		8 (10.0%)	
Subtype of ALL (No. =40)	B-ALL	8 (100.0%)	24 (37.5%)	0.264
	T-ALL	0 (0.0%)	8 (12.5%)	
HSM	No	4 (50.0%)	40 (55.6%)	0.764
	Yes	4 (50.0%)	32 (44.4%)	
Cytogenetic FISH	Normal Karyotyping	4 (50.0%)	36 (50.0%)	1.000
	t (9;22) (q34; q11)	2 (25.0%)	14 (19.5%)	0.624
	t (8;21) (q22; q22)	0 (0.0%)	8 (11.1%)	0.320
	RARA rearrangement	0 (0.0%)	8 (11.1%)	0.320
	t (21;21)	0 (0.0%)	2 (2.8%)	0.633
	Abnormal Karyotyping	2 (25.0%)	2 (2.8%)	0.8
	11q23 (MLL) rearrangement	0 (0.0%)	2 (2.8%)	0.004
CHR on day 28	Not in remission In remission	4(50.0%) 4 (50.0%)	48 (66.7%) 24 (33.3%)	0.457
TLC (10^9^/L)	Median (IQR)	19.8 (8.75–38.8)	11.4 (6.1–60)	0.928
	Range	5.5–50	1–516.1	
Hb (g/dl)	Mean ± SD	6.95 ± 1.87	8.16 ± 1.95	0.245
	Range	5.1–9.5	5–13.8	
Platelets (10^9^/L)	Median (IQR)	32.5 (16.5–51.5)	48 (27.5–93)	0.241
	Range	11–60	10–274	
PB blast (%)	Mean ± SD	69.75 ± 17.52	52.28 ± 23.94	0.166
	Range	45–84	5–96	
BM blasts (%)	Mean ± SD	88.75 ± 6.29	75.03 ± 23.42	0.255
	Range	80–95	24–98	
CD34 (%)	Median (IQR)	0.7 (0.3–1)	62 (3–84.5)	0.007
	Range	0.2–1	0–100	
CD34-positive patients (No.)	Negative	8 (100.0%)	28 (38.9%)	0.020
	Positive	0 (0.0%)	44 (61.1%)	
HLA-DR (%)	Median (IQR)	1 (1–1)	1 (1–88)	0.103
	Range	1–1	1–98	
HLA-DR-positive patients (No.)	Negative	8 (100.0%)	44 (61.1%)	0.122
	Positive	0 (0.0%)	28 (38.9%)	
TDT (%)	Median (IQR)	1 (1–1)	1 (1–40.5)	0.214
	Range	1–1	1–92	
TDT-positive patients (No.)	Negative	8 (100.0%)	64 (88.9%)	0.197
	Positive	0 (0.0%)	8 (11.1%)	
CD117 (%)	Median (IQR)	1 (0.55–1)	39.5 (1–87.5)	0.051
	Range	0.1–1	0–98	
CD117-positive patients (No.)	Negative	8 (100.0%)	32 (44.4%)	0.035
	Positive	0 (0.0%)	40 (55.6%)	
MPO (%)	Median (IQR)	1 (1–1)	47.5 (1–96.5)	0.025
	Range	1–1	1–99.7	
MPO-positive patients (No.)	Negative	8 (100.0%)	32 (44.4%)	0.035
	Positive	0 (0.0%)	40 (55.6%)	

*P*-value > 0.05: Non-significant; *P*-value < 0.05: Significant; *P*-value < 0.01: Highly significant

*: Chi-square test; ≠: Mann-Whitney test

**Table 4:** Comparison between the RECAF negative (< 13.31%) vs. positive (≥ 13.31%) ALL patients

ALL patient groups (No = 40)		RECAF%		P-value
		< 13.31%	≥ 13.31%	
		No. = 8	No. = 32	
Age (years)	Median (IQR)	11.5 (8–13.5)	5 (3–14)	0.442
	Range	6–14	1–15	
Sex	Female	2 (25.0%)	10 (31.25%)	0.807
	Male	6 (75.0%)	22 (68.75%)	
Subtype of ALL	B-ALL	8 (100.0%)	24 (75.0%)	0.264
	T-ALL	0 (0.0%)	8 (25.0%)	
HSM	No	4 (50.0%)	10 (31.3%)	0.320
	Yes	4 (50.0%)	22 (68.8%)	
Cytogenetic FISH	Normal Karyotyping	4 (50.0%)	18 (56.3%)	0.751
	t (9;22) (q34; q11)	2 (25.0%)	10 (31.3%)	0.638
	t (21;21)	0 (0.0%)	2 (6.3%)	0.468
	Abnormal Karyotyping	2 (25.0%)	0 (0.0%)	0.201
	11q23 (MLL) rearrangement	**0 (0.0%)**	**2 (6.3%)**	**0.004**
TLC (10^9^/L)	Median (IQR)	19.8 (8.75–38.8)	12.8 (5.15–43.4)	0.777
	Range	5.5–50	2.3–516.1	
Hb(g/dl)	Mean ± SD	6.95 ± 1.87	8.68 ± 1.77	0.101
	Range	5.1–9.5	5.1–11.6	
Platelets(10^9^/L)	Median (IQR)	32.5 (16.5–51.5)	47 (27.5–57)	0.277
	Range	11–60	20–199	
PB blast (%)	Mean ± SD	69.75 ± 17.52	57.5 ± 24.67	0.366
	Range	45–84	14–96	
BM blasts (%)	Mean ± SD	88.75 ± 6.29	86.38 ± 16.11	0.779
	Range	80–95	50–98	
CD34 (%)	Median (IQR)	**0.7 (0.3–1)**	**71 (4.5–82.5)**	**0.014**
	Range	**0.2–1**	**0.1–99**	
CD34-positive patients (No.)	Negative	**8 (100.0%)**	**10 (31.3%)**	**0.013**
	Positive	**0 (0.0%)**	**22 (68.8%)**	
CD19 (%)	Median (IQR)	95.5 (92.5–97.5) 91–98	95 (1–98) 0–99	0.739
	Range			
CD19-positive patients (No.)	Negative	0 (0.0%) 8 (100.0%)	8 (25%) 24 (75%)	0.197
	Positive			
CD79a (%)	Median (IQR)	97 (49–98) 1–99	87 (1–98.5) 1–100	0.598
	Range			
CD79a-positive patients (No.)	Negative	0 (0.0%) 8 (100.0%)	10 (31.3%) 22 (68.8%)	0.197
	Positive			
CD10 (%)	Median (IQR)	95 (92.5–97)	80.5 (5.5–99)	0.702
	Range	92–97	1–99	
CD10-positive patients (No.)	Negative	0 (0.0%)	10 (31.3%)	0.197
	Positive	8 (100.0%)	22 (68.8%)	
CD20 (%)	Median (IQR)	0.6 (0.1–2.5)	1 (1–6.5)	0.130
	Range	0–4	0–80	
CD20-positive patients (No.)	Negative	8 (100.0%)	28 (87.5%)	0.456
	Positive	0 (0.0%)	4 (12.5%)	
CD3 (%)	Mean ± SD	1.1 (1–1.6)	1 (1–26)	0.171
	Range	1–2	0–100	
CD3-positive patients (No.)	Negative	**8(100.0%)**	24 (75.0%)	0.264
	Positive	0 (0.0%)	8 (25.0%)	
CD7 (%)	Median (IQR)	1.1 (1–1.6)	1 (1–26)	0.842
	Range	1–2	0–100	
CD7-positive patients (No.)	Negative	8 (100.0%)	24 (75.0%)	0.264
	Positive	0 (0.0%)	8 (25.0%)	
CD2 (%)	Median (IQR)	0.55 (0.05–1)	1 (1–39)	0.171
	Range	0–1	0–100	
CD2-positive patients (No.)	Negative	8 (100.0%)	24 (75.0%)	0.264
	Positive	0 (0.0%)	8 (25.0%)	
CD5 (%)	Median (IQR)	0.85 (0.35–1.5)	1 (1–6.5)	0.300
	Range	0–2	0–100	
CD5-positive patients (No.)	Negative	8 (100.0%)	26 (81.3%)	0.348
	Positive	0 (0.0%)	6 (18.8%)	

*P*-value > 0.05: Non-significant; *P*-value < 0.05: Significant; *P*-value < 0.01: Highly significant. *: Chi-square test; ≠: Mann-Whitney test, Significant values are provided in bold

## Discussion

Acute leukemia (AL) stands as the leading cause of cancer-related deaths among children, constituting one-third of all cancer cases, with an alarmingly increasing incidence each year [19]. Remarkably, acute ALL accounts for approximately 80% of these cases, representing a substantial increase relative to adult populations, whereas AML is predominantly seen in adults [4, 20, 21]. 

The gold standard for diagnosing childhood leukemia relies on the morphological evaluation of BM aspirates. Complementing this, FCI is crucial for identifying the leukemic cell population and determining vital prognostic subgroups [7, 22, 23]. 

The recent detection of re-expressed AFP receptor (RECAF) in various cancer patients strongly supports the hypothesis that receptor-mediated endocytosis plays a crucial role in the pathogenesis of these malignancies [24]. Numerous studies have demonstrated that RECAF is re-expressed in poorly differentiated malignant cells across various types of cancer, including hepatocellular carcinoma (HCC), breast cancer, colorectal cancer, rectal cancer, and lung cancer [16, 17]. These studies indicate that the interaction mediated by RECAF is linked to malignant proliferation, poor differentiation, and the eventual progression of the disease [8, 16]. Emerging evidence increasingly supports the use of RECAF as a robust marker with diverse applications in these malignancies. Additionally, its promising role in targeting and treating cancer cells via endocytosis could pave the way for advances in cancer management [16, 17, 25]. 

Our study aimed to evaluate RECAF expression using FCI in 80 pediatric patients diagnosed with de novo AML and ALL. This was compared with 40 age- and sex-matched control subjects without hematological malignancies to determine the clinical utility of RECAF. All patients with leukemia were diagnosed using morphological criteria, immunophenotyping (IPT), and cytogenetic analysis, in accordance with the WHO-HAEM5 and ICC guidelines [26, 27]. Our AL patients were monitored on day 28 after initiation of induction therapy, and CHR was assessed. In our study, the most common AML FAB subtypes were M1 and M2, followed by M4/M5, M3, and M0, as determined by flow cytometry and cytogenetic studies. Across all FAB subtypes in our study, B-ALL was the most common, followed by T-ALL, as determined by flow cytometry and cytogenetic studies.

In our study, we aimed to establish RECAF as a marker for malignancy and leukemia. We observed that RECAF expression was significantly higher in all patients with AL (leukemic blasts) than in the control group (normal blasts/hematogones), and this increase correlated with blast cell count. These results suggest that RECAF expression could be a groundbreaking marker for pediatric MRD. We also identified a cutoff of ≥ 13.31% for positive RECAF expression in AL patients (AML and ALL) compared with controls. This threshold was used to categorize AL patients into RECAF-positive and RECAF-negative groups. The low RECAF levels in the control group, all below 13.3%, suggest minimal implications. This expression may be due to activated or stimulated T cells, which current research supports. Importantly, normal mature adult tissues typically do not express RECAF. This finding highlights the importance of further exploring the biological mechanisms behind RECAF expression, which may shed new light on cellular differentiation and immune responses [8, 28]. 

Sedky et al. (2020) investigated RECAF expression using immunohistochemistry on archival bone marrow trephine biopsies in a related study. They found that the percentage of RECAF-positive cells was significantly higher in all ALL cases than in the non-malignant control. The researchers found notably elevated RECAF expression in patients with B-NHL and CLL compared with non-malignant controls [16]. The researchers interpreted their findings as evidence that RECAF expression reflects an abnormal, malignant, or leukemic maturation pattern rather than immaturity. Furthermore, they observed negative RECAF expression in malignant cases arising from partially differentiated or end-stage cells, including myeloma cells and those with chronic myeloid leukemia (CML) [16]. Other studies have similarly demonstrated this by using anti-RECAF monoclonal antibodies for immunofluorescence and immunohistochemistry on paraffin sections of both benign and malignant tumor tissues. These studies reported positive staining in human mammary carcinomas, lung cancers, colon carcinomas, and other malignancies. In contrast, benign adenomas showed no RECAF staining [29]. 

Our study also examined the pattern of RECAF expression in cases of acute leukemia (AL). Notably, all tested cases of AML and T-ALL exhibited positive RECAF expression. Additionally, our research revealed a positive correlation between RECAF and the expressions of CD64, CD11c, and CD13. In contrast, B-ALL cases showed mixed results, with eight patients testing negative for RECAF. All eight RECAF-negative B-ALL cases were negative for several markers, including CD34, TdT, HLA-DR, and CD20; however, they were positive for CD19, CD79a, and CD10. The diagnosis for these RECAF-negative cases was primarily based on morphological analysis and the presence of immature cells within the CD45 dim (blast) gate.

The presence of RECAF-negative B-ALL cases strongly indicates a more advanced stage of blast maturation, as evidenced by the striking absence of most immaturity markers. Moreover, the consistent negativity of both RECAF and CD34 across all eight cases underscores a significant relationship between RECAF expression and cellular immaturity. Such a correlation highlights the potential of RECAF as a crucial marker for evaluating blast differentiation in B-ALL, offering new insights for clinical assessment and treatment strategies.

Another assumption is that the notable positivity observed in most of our studied AL cases suggests that RECAF could be an effective flow cytometric marker for monitoring minimal residual disease (MRD), as it shows negative expression in normal hematogones, as shown in our control group. This is especially relevant when critical lineage-differentiating markers may be lost, as in CD19-depleted relapsed B-ALL cases following CD19-targeted CAR-T therapy [30]. This consideration extends to AML cases that may initially present without a clear identification of their maturity stage (CD34-negative AML) or a distinct leukemic-associated immunophenotype (LAIP), notably in instances like acute promyelocytic leukemia (APL) and other subtypes of AML (non-APL AML), particularly when there is a significant presence of dysplastic populations [31]. Nonetheless, conducting further research with larger sample sizes would be beneficial to substantiate these findings.

Numerous studies are investigating the potential of RECAF as a non-specific, widespread cancer biomarker for detecting malignancy in tissue sections, blood smears, and body fluids [8–29]. A 2012 study by Moro et al. demonstrated that a RECAF immunoassay can effectively differentiate between breast cancer tissue and that from normal subjects or benign breast diseases. One of the most significant findings of this study is that the RECAF assay can detect early-stage breast cancer with high sensitivity and specificity, indicating that RECAF could be used for tumor screening in the future [28]. In a similar context, Sedky et al. (2020) reported that RECAF exhibits distinct expression patterns in patients with B-NHL/CLL. Its unique pattern may be related to differences in cell maturation stages among these malignancies (immunoblasts vs. immunocytes). They suggested it as a promising tool for diagnosing and monitoring MRD in different subtypes of B-NHL [16]. 

Moreover, researchers believe that the expression of RECAF on immature and malignant cells positions it as a promising target for future therapies, particularly given its receptor-mediated endocytic nature [17]. Various complexes combining AFP with different toxic substances are currently being tested to validate the use of the RECAF-AFP interaction for drug and toxin delivery in the treatment of various malignant diseases [25]. Recently, an oral anticancer drug has been developed to target RECAF. This drug is designed as a non-covalent AFP complex and has attracted substantial interest for its ability to induce apoptosis in tumor cells via selective AFP-receptor-mediated endocytosis. The formulation incorporates AFP as the carrier and Atractyloside as the agent that induces apoptosis [17, 32].

Regarding the prognostic utility of RECAF in our study, there was no difference in complete hematological remission (CHR) at day 28 between RECAF-positive and RECAF-negative AL patients, nor between RECAF-positive and RECAF-negative ALL patients. These findings negate RECAF’s prognostic value in AL patients. In agreement with our findings, many studies confirmed that the combination of RECAF with other markers (for instance, RECAF and CA125 in ovarian cancer) provides the specificity and sensitivity necessary to screen for cancer, mainly to detect early stages of the disease; however, they found no prognostic evidence of RECAF in disease progression [18, 25, 33, 34]. 

## Conclusion

This study is the first to explore RECAF expression in pediatric acute leukemia patients using flow cytometry. Our data show significantly higher RECAF levels in patients with acute leukemia compared to non-leukemic controls, supporting the idea that RECAF expression helps differentiate leukemic blasts from normal hematogones and cells. These findings suggest that RECAF could serve as a new biomarker for early detection of leukemic blasts and for monitoring minimal residual disease (MRD) in AL patients. We also recommend adding RECAF measurement to routine diagnostic procedures for AL cases lacking specific lineage or immaturity markers or LAIP, to enhance follow-up precision and treatment management.

However, our study has limitations, including a small sample size, a single-center design, and the absence of RECAF assessment after induction therapy and exploration of RECAF by genotype. Further research is needed to evaluate the clinical usefulness of RECAF in blood cancers. Future studies should include different types of leukemia, a broader age range, and more detailed flow cytometry, chromosomal, and molecular analyses. Additionally, long-term follow-up is crucial to determine RECAF’s impact on overall survival (OS) and disease-free survival (DFS).

## Supplementary Information

## Data Availability

The datasets used and/or analyzed during the current study are available from the corresponding author on reasonable request. On https://drive.google.com/file/d/1bSGmDccUl2i8PuFD_9Qe3ksuvTy8Zijg/view?usp=sharing, data sharing applies to this article, as datasets were generated and analyzed during the current study.

## Abbreviations

AFP: Alpha-fetoprotein
RECAF: Receptors for AFP
AL: Acute leukemia
ALL: Acute lymphoblastic leukemia
AML: Acute myeloid leukemia
B-NHL: B non-Hodgkin lymphoma
CLL: Chronic lymphocytic leukemia
CBC: Complete blood count
BM: Bone marrow
PB: Peripheral blood
LDH: Lactate dehydrogenase
TLC: Total leukocytic count
IPT: Immunophenotyping
CNS: Central nervous system
FISH: Fluorescence in situ hybridization
FCI: Flow cytometric immunophenotyping
CHR: Complete Hematological Remission
LAIP: Leukemia-associated immunophenotype
MRD: Minimal residual disease
CML: Chronic myeloid leukemia
APL: Acute promyelocytic leukemia
