# Supplementary material for: Pilot study on RECAF (Receptor for Alpha-Fetoprotein) expression in pediatric acute leukemia: insights from flow cytometry analysis
Source: J Egypt Natl Canc Inst. 2026 Sep 2;38:66. doi: 10.1186/s43046-026-00404-4 (PMC13538328; doi:10.1186/s43046-026-00404-4)
Supplement: Supplementary file 1 [file 43046_2026_404_MOESM1_ESM.docx]

**Manuscript Title:**

**Pilot Study on RECAF (Receptor for Alpha-Fetoprotein) Expression in Pediatric Acute Leukemia: Insights from Flow Cytometry Analysis.**

**Table 1:** **Describing and comparing Demographics, Lab data, and cytogenetic characteristics between the Control and patient group:**

| **All groups** | | **Control group** | **Patients group** | **P-value** |
| --- | --- | --- | --- | --- |
|  |  | **No. = 40** | **No. = 80** |  |
| **Age (years)** | **Median (IQR)** | 12 (7 – 13) | 11 (5.5 – 14) | 0.653 |
|  | **Range** | 2 – 13 | 1 – 17 |  |
| **Sex** | **Female** | 16 (40.0%) | 26 (32.5%) | 0.654 |
|  | **Male** | 24 (60.0%) | 54 (67.5%) |  |
| **FAB subtype**  **for AML**  **(No. =40)** | **M0**  **M1/M2**  **M4/M5**  **M3** |  | 2 (2.5%)  20 (25%)  10 (12.5%)  8 (10.0%) | − |
| **FAB subtype**  **for ALL**  **(No. =40)** | **B – ALL**  **T – ALL** |  | 32 (40%)  8 (10.0%) | − |
| **HSM** | No | 36(90.0%) | 44 (55.0%) | 0.000 |
|  | Yes | 4 (10.0%) | 36 (45%) |  |
| **Cytogenetic FISH** | Normal Karyotyping |  | 40 (50.0%) | _ |
|  | t (9;22) (q34; q11) |  | 16 (20.0%) |  |
|  | t (8;21) (q22; q22) |  | 8 (10.0%) |  |
|  | RARA rearrangement |  | 8 (10.0%) |  |
|  | t (21;21) |  | 2 (2.5%) |  |
|  | 11q23 (MLL) rearrangement |  | 2 (2.5%) |  |
|  | Abnormal Karyotyping |  | 4 (5%) |  |
| **TLC (10^9^/L)** | **Median (IQR)**  **Range** | 7 (6 – 11)  5 – 27 | 12 (6 – 55)  1 – 516 | 0.129 |
| **Hb (g/dl)** | **Mean ± SD**  **Range** | 12.52 ± 1.03  10.7 – 14.4 | 8.04 ± 1.95  5 – 13.8 | 0.000 |
| **Platelets (10^9^/L)** | **Median (IQR)**  **Range** | 31 (18 – 63)  2 – 85 | 47 (27 – 78)  10 – 274 | 0.220 |
| **PB blast (%)** | **Mean ± SD**  **Range** | 0 ± 0  0 – 0 | 54.02 ± 23.79  5 – 96 | **0.000** |
| **BM blasts (%)** | **Mean ± SD**  **Range** | 1.95 ± 0.83  1 – 4 | 76.4 ± 22.64  24 – 98 | 0.000 |
| **RECAF %** | **Median (IQR)**  **Range** | 6 (4 – 11) | 40 (28 – 76) | 0.000 |
|  |  | 0 – 13 | 1 – 100 |  |
| **MFI – RECAF** | **Median (IQR)**  **Range** | 6 (2 – 10) | 6 (4 – 7) | 0.942 |
|  |  | 0 – 12 | 1 – 12 |  |

P-value > 0.05: Non-significant; P-value < 0.05: Significant; P-value < 0.01: Highly significant *: Chi-square test; •: Independent t-test; ≠: Mann-Whitney test.

**Table 2:** **Comparison of RECAF expression on blast cells between the Control and Patient groups and 0between the AML and ALL groups:**

|  | | **Control group** | **Patients group** | **P-value** | **AML** | **ALL** | **P-value** |
| --- | --- | --- | --- | --- | --- | --- | --- |
|  |  | **No. = 40** | **No. = 80** |  | **No. = 40** | **No. = 40** |  |
| **RECAF %** | **Median (IQR)** | **6 (4 – 11)** | **40 (28 – 76)** | **0.000** | 47.23  (32.43 – 82.1) | 36.53  (21.14 – 68.14) | 0.117 |
|  | **Range** | **0 – 13** | **1 – 100** |  | 22.81 – 97.47 | 1.2 – 99.7 |  |
| **MFI-RECAF** | **Median (IQR)** | 6 (2 – 10) | 6 (4 – 7) | 0.942 | **6.87**  **(5.52 – 7.32)** | **4.72**  **(3.41 – 6.05)** | **0.011** |
|  | **Range** | 0 – 12 | 1 – 12 |  | **3.6 – 11.78** | 1.24 **– 11.3** |  |

**Table 3: Multivariate linear regression analysis for factors associated with RECAF% among the studied patients**

|  | **Unstandardized  Coefficients** | | **Standardized  Coefficients** | **t** | **P-value** |
| --- | --- | --- | --- | --- | --- |
|  | **B** | **SE** | **Beta** |  |  |
| **(Constant)** | **36.230** | **6.398** |  | **5.663** | **0.000** |
| **CD13** | **0.223** | **0.104** | **0.314** | **2.133** | **0.040** |
| **CD64** | **0.026** | **0.207** | **0.030** | **0.125** | **0.901** |
| **CD11C** | **0.365** | **0.279** | **0.280** | **1.310** | **0.198** |

**Multivariate linear regression analysis identified CD13 as the only independent factor significantly associated with RECAF%, demonstrating a positive association (β = 0.314, B = 0.223, p = 0.040). In contrast, neither CD64 (β = 0.030, B = 0.026, p = 0.901) nor CD11c (β = 0.280, B = 0.365, p = 0.198) showed a statistically significant association with RECAF%.**

**Table 4: Multivariate linear regression analysis for factors associated with RECAF MFI among the studied patients**

|  | **Unstandardized  Coefficients** | | **Standardized  Coefficients** | **t** | **Sig.** |
| --- | --- | --- | --- | --- | --- |
|  | **B** | **SE** | **Beta** |  |  |
| **(Constant)** | **6.334** | **1.656** |  | **3.826** | **0.001** |
| **CD117** | **0.017** | **0.009** | **0.305** | **2.077** | **0.041** |
| **MPO** | **0.006** | **0.016** | **0.117** | **0.375** | **0.710** |
| **CD64** | **0.018** | **0.015** | **0.250** | **1.214** | **0.233** |
| **BM blasts%** | **-0.016** | **0.018** | **-0.152** | **-0.876** | **0.387** |

**Multivariate linear regression analysis identified CD117 as the only independent factor significantly associated with RECAF MFI, showing a positive association (β = 0.305, B = 0.017, p = 0.041). In contrast, MPO (β = 0.117, B = 0.006, p = 0.710), CD64 (β = 0.250, B = 0.018, p = 0.233), and bone marrow blasts percentage (β = −0.152, B = −0.016, p = 0.387) were not significantly associated with RECAF MFI.**

**Table 5:** Demonstrating the relationship between CD34 and RECAF expression in Acute leukemia patients:

| **Acute leukemia patients (No =80)** | **RECAF positive (No =72)** | **RECAF negative (No =8)** |
| --- | --- | --- |
| **CD34 positive** | **44 (100.0%)** | 0 (0.0%) |
| **CD34 negative** | 28 (77.8%) | **8 (22.2%)** |
